# Gynecomastia: The 4Dx technique

**DOI:** 10.1055/s-0044-1800779

**Published:** 2024-12-12

**Authors:** Aakanksha Goel, Sudhanshu Punia, Amit Gupta

**Affiliations:** 1 Department of Plastic Surgery, Divine Aesthetic Surgery, New Delhi, India

**Keywords:** gynecomastia, liposuction

## Abstract

In surgery for gynecomastia, it is not sufficient to just remove the gland or do a liposuction that addresses the front of the chest only as it is not aesthetically pleasing for the patient and the surgeon alike. Most patients expect to achieve a sculpted look, which includes not only the breast area but also the surrounding areas such as the sides, the axillae, and the infraclavicular region. To tackle these areas and achieve a well-sculpted and aesthetic look, we describe the 4Dx (4 Directions) technique of liposuction for gynecomastia. It also makes the procedure more objective for plastic surgeons while allowing for individualization of the procedure.

## Introduction

Surgery for gynecomastia is the third most common aesthetic procedure done in men worldwide. 1 With growing self-consciousness, it is no longer enough for the patients to just get the gland removed or do a liposuction, which addresses only the front of the chest. We believe that there are three main considerations while doing any liposuction for gynecomastia: (1) regions that need to be addressed, (2) port sites that allow efficient liposuction in the regions, preferably in two different directions perpendicular to each other, while leaving minimum scars, and (3) how much liposuction should be done in the specific areas, that is, which regions to make thin and which ones to leave thicker than others.

## Idea

Most patients for gynecomastia surgery expect to have a sculpted look, which includes not only the breast area but also the surrounding areas such as the sides, the axilla, and the infraclavicular region. This prompted us to address the complete chest so that an aesthetically pleasing look can be achieved. To tackle the above considerations and make it more objective for plastic surgeons, we described the 4Dx (4 Directions) technique of liposuction in gynecomastia.

From May 2020 till September 2023, we operated on a total of 2,183 gynecomastia cases, aged 16 to 65 years. These included 108 grade 1 (1a-25; 1b-83), 937 grade 2 (2a-831; 2b-106), 1,003 grade 3 (3a-638; 3b-365), and 135 grade 4 (4a-62; 4b-73) as per Punia and Gupta classification. 2 We used the 4Dx technique for all grade 2, 3, and 4 cases (both “a” and “b” subgroups).

**Fig. 1:**
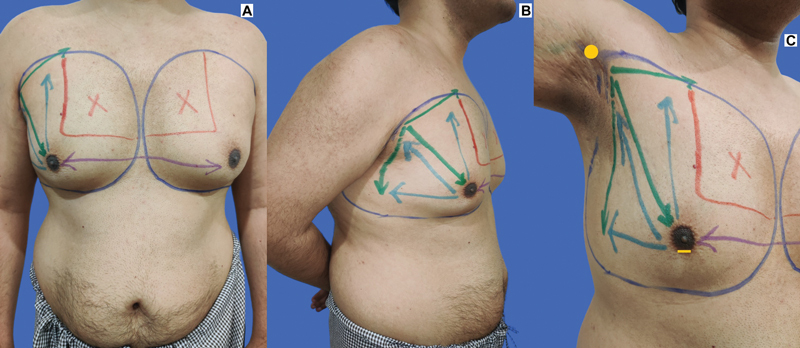
( **A** and **B** ) Markings of the regions to be addressed (dark blue marker). “Bra-like” appearance of the marking for liposuction is noted. ( **C** ) Site of axillary and infra-areolar incisions (yellow marker); direction of strokes of the liposuction cannula from axillary incision (green arrows); from the infra-areolar incision (light blue arrows); cross-tunneling to opposite side (purple arrows) and upper inner quadrant (red outline) are marked.

The patient is examined for areas of fullness, which need liposuction, extent of gland on palpation and dynamic changes in shape of the chest with contraction and relaxation of the pectoralis muscle, and any skeletal abnormality. Identification of the regions of concern is followed by marking of the areas (marked in dark blue in Fig. 1 ). Two incisions are marked, one just behind the anterior axillary fold at the apex so that it is hidden within the axilla, 2 to 4 mm in length. The other incision is at the 6 o'clock position of the nipple-areola complex (NAC) (at the junction of pigmented NAC with lighter surrounding skin), 2 to 4 mm in length (incision sites marked in yellow in Fig. 1C ). Following general anesthesia, infiltration using a tumescent fluid of choice is done in the superficial and deep planes in all the regions as marked followed by a waiting period of 7 to 10 minutes. If any energy-based devices such as VASER/radiofrequency must be used, it may be done at this point. Finally, the cannula is inserted in four different directions for liposuction, viz., infraclavicular, prepectoral, axillary fold, and lateral chest wall ( Table 1 ). Infiltration may also be done along the same directions for liposuction.

**Table 1:** Description of direction of liposuction and the respective liposuction cannulae

Direction of liposuction	Color code in Fig. 1	Liposuction cannulae (diameter, length)
From axillary incision to lateral chest wall, prepectoral area (including inframammary fold [IMF] disruption) and infraclavicular area	Green	4 mm or 5 mm, 25–30 cm
From infra-areolar incision to the axilla, anterior axillary fold, infraclavicular area (upper outer quadrant), and the lateral chest wall	Light blue	4 mm, 20–25 cm
Cross-tunneling from the infra-areolar incision to the opposite side prepectoral and IMF area	Purple	4 mm, 30–35 cm
None to little liposuction in the upper inner quadrant via axillary incision	Red	3 mm, 25 cm (feathering only)

**Fig. 2:**
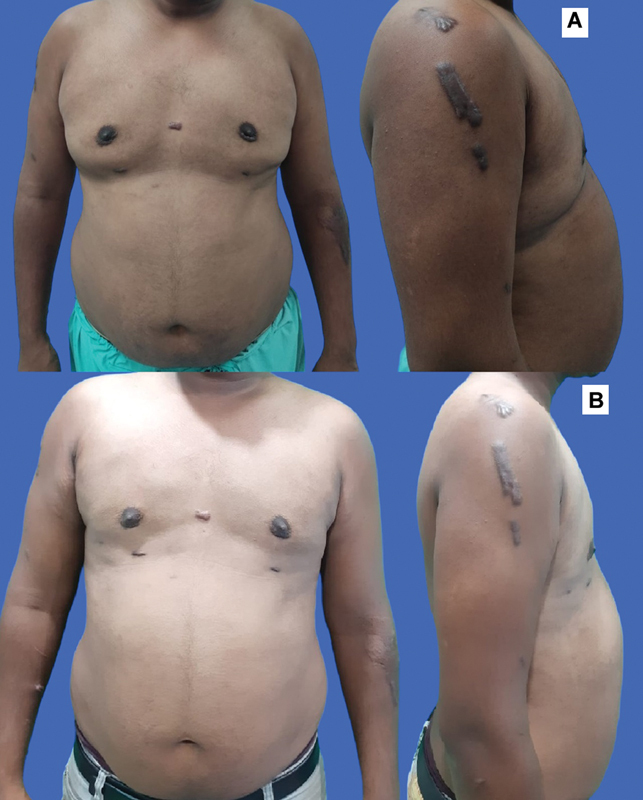
( **A** ) A case of gynecomastia operated elsewhere, liposuction of the prepectoral area and gland removal done. Please note the prominent scars and the wide and flat unnatural appearance of the chest, heaviness in the lateral side rolls. ( **B** ) After 4Dx liposuction, well-defined contours seen.

Liposuction from the axillary incision is done in three directions, namely, the lateral chest wall or side rolls, the prepectoral area or front of chest, and the infraclavicular area. The rationale of doing liposuction on the side rolls area is to prevent a flat and wide look of the chest ( Fig. 2A ). Most patients expect to see pectoral contouring on the side, which can only be achieved by liposuction in this area. This region can be suctioned well and made relatively thin without causing any contour deformity ( Fig. 2B ). From the infra-areolar incision, the axilla, anterior axillary fold, infraclavicular area (upper outer quadrant only), and the lateral chest wall is addressed. This ensures no residual fullness in the axilla, which appears more prominent after surgery and causes distress to the patient if not dealt with. Lastly, cross-tunneling from the infra-areolar incision to the opposite chest is done in the prepectoral and inframammary fold (IMF) area to disrupt it further, while being careful to tunnel in a plane superficial to the sternum and the cannula palpable at all times. This helps in tackling the main breast area, especially the area between the NAC and IMF from two different directions, which is needed specially if the gland is bulky and prevents adequate liposuction from one direction. However, this region must not be made too thin as it can lead to a depression in the standing position postoperatively. Sufficient tissue must be left to support the infra-areolar region. The upper inner quadrant of the chest is where the bulk of the pectoralis major muscle is. Patients desire the appearance of a well-developed pectoral muscle as it gives an “athletic look” to them. Thus, this is the region which is not suctioned at all or just a few strokes are made with the cannula to smoothen out to the contours. More importantly, as the blood supply of the NAC is derived from the lateral and internal thoracic artery and their perforators, minimal suctioning in this area helps preserve vascularity (while not compromising with the contour), especially in the more severe grades of gynecomastia. 3 However, the differential thickness of different zones (measured subjectively with skin pinch by the surgeon) is based on the patient's physique and grade of gynecomastia and cannot be standardized objectively.

This technique ensures that all the regions are suctioned from two directions, ensuring that the liposuction is complete and natural contouring is achieved. The gland must be excised from the infra-areolar incision after the complete liposuction and contouring is achieved, while leaving a small disc of gland (∼0.5 cm) beneath the NAC to prevent a crater deformity. One should never make the area immediately lateral or superior to the NAC too thin to avoid a depression deformity. It needs gentle liposuction with thin cannulas or just smoothening out the gland with scissors. The final outcomes, assessed by clinical examination and photographic evaluation, reveal an effective contouring ( Fig. 2 ).

## Discussion

Several different techniques have been described for liposuction using different port sites. The large number of publications is testimony to the fact that none is perfect. Most of them do not describe the direction of liposuction for an efficient contouring. We aimed to simplify the procedure and make it more objective for use by all plastic surgeons.

**Fig. 3:**
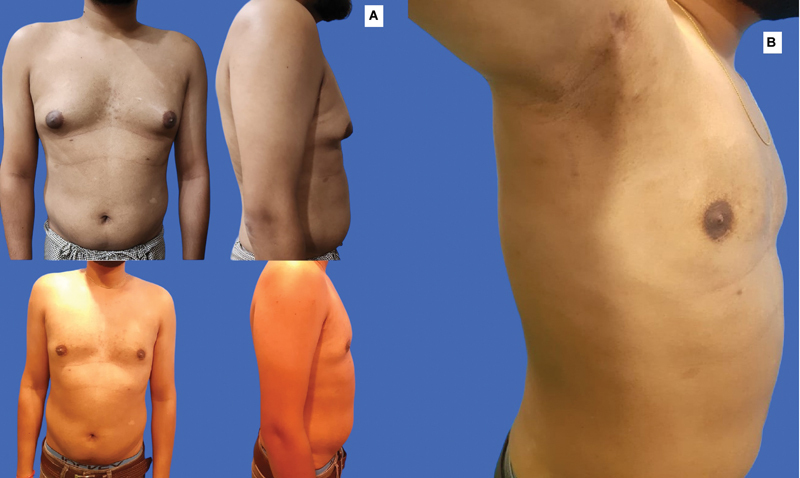
( **A** ) Preoperative (above) and postoperative (below) photographs of a patient with gynecomastia operated with the 4Dx technique. ( **B** ) Stealth incisions (axilla and infra-areolar) do not leave visible scars.

It is important to note the differences between a patient undergoing high-definition sculpting versus a 4Dx gynecomastia liposuction. The body type of the patient who desires high definition with chest packs is lean and muscular with minimal fat component. The negative and positive spaces are created to give a chiseled look. 4Dx gynecomastia surgery is for regular men ( Fig. 3 ) who may or may not have well-developed muscles and thus high-definition liposuction may not be suitable for them. Thus, 4Dx liposuction fashions a look that matches their overall body habitus.

**Fig. 4:**
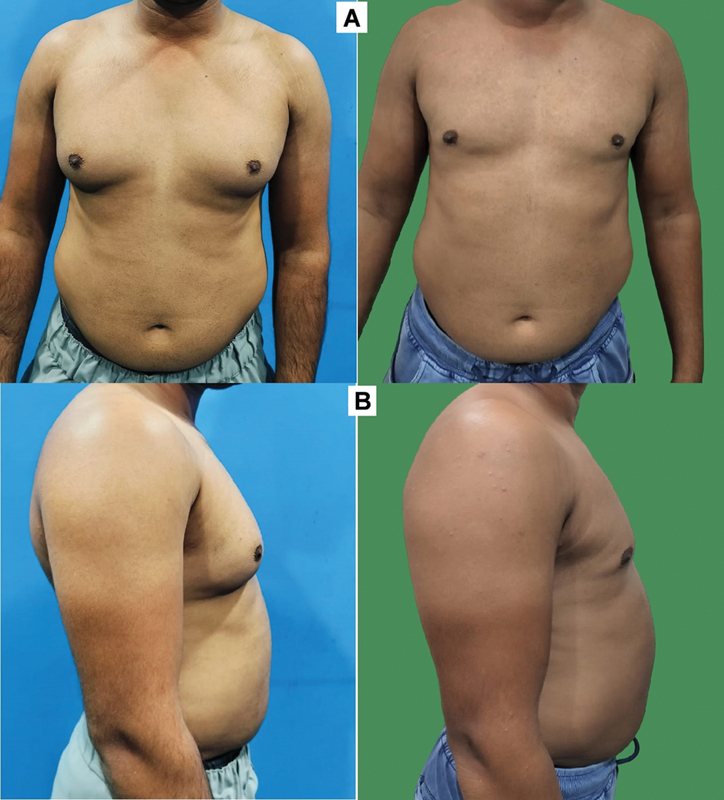
Preoperative (left) and postoperative (right) photographs of a patient who has side rolls (grade 3a). ( **A** ) Front views. ( **B** ) Side views. A well-contoured shape of the chest achieved with the 4Dx technique.

The authors believe that even the slightest scars are unacceptable in gynecomastia surgery. Thus, stealth incisions such as in the anterior axillary fold and infra-areolar incision at the junction of pigmented NAC with lighter surrounding skin are most appropriate ( Fig. 3B ). 4 Even with respect to vascularity, infra-areolar incision appears to be the best. 3 However, many authors give incisions in the lateral and inferior chest. The published photographs speak for themselves. The scars are evident. 5 6 The use of single axillary incision (around 3 cm in length to facilitate excision of the gland) has been described in literature with use of special equipment and expertise, such as CO _2_ insufflators, vessel sealing devices, endoscopic equipment, etc. Apart from adding to the cost of the procedure, a longer scar would be more conspicuous in Indian skin types and hence unacceptable to the patients. 7 We agree with other authors who have also felt the need to address the side rolls or divide the chest into aesthetic units as just addressing the front of the chest or breast region is not enough ( Fig. 4 ). 8 9

**Fig. 5:**
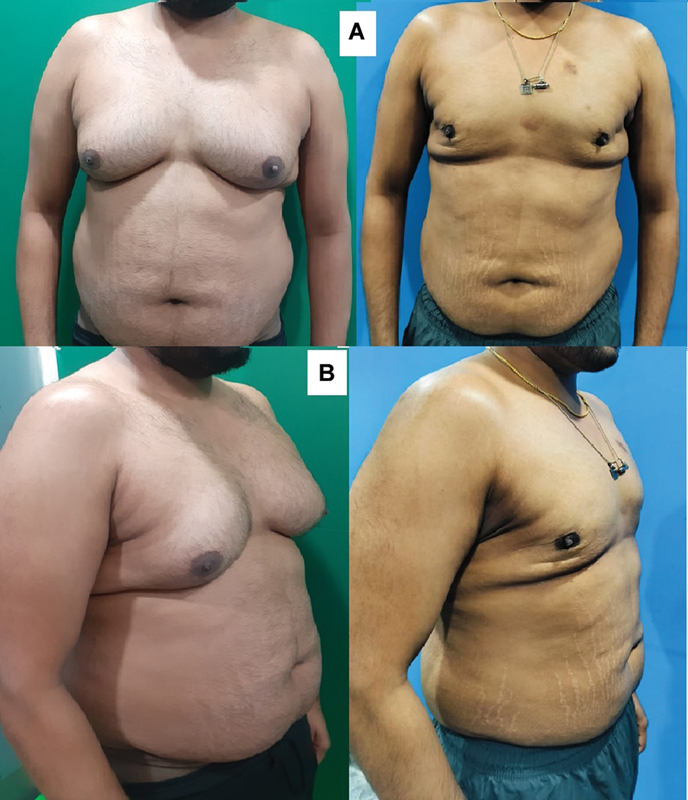
Preoperative and postoperative photographs of a case of gynecomastia grade 4b. ( **A** ) Front views. ( **B** ) Oblique views. Even after liposuction and gland removal, along with a lift for the nipple-areola complex (NAC), such patients have excess skin persistent in the lower part of the chest, which may be excised in a second stage as per the desires of the patient.

This technique allows for individual modifications to suit surgeons' preference and the needs of the patient. The tumescent fluid for infiltration is as per surgeons' choice. Use of energy-based devices such as VASER- or radiofrequency-assisted liposuction can be done prior to the 4Dx technique of liposuction for their described advantages. The authors used VASER in a subset of their patients, especially those with skin laxity. In case of ptosis, crescentic supra-areolar skin excision can be planned along with the 4Dx technique, as was done by the authors for subgrade “b” of each grade of gynecomastia ( Fig. 5 ). Although the authors only put closed-suction drains electively in grade 4b gynecomastia, surgeons can choose to place drains as per their practice through the axillary incision itself, without the need for an extra incision, thus preventing more scars. 10

**Fig. 6:**
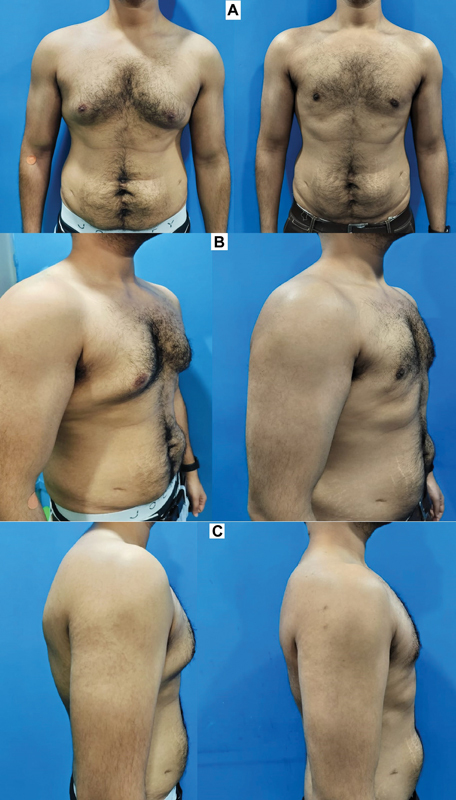
A case of gynecomastia grade 2a, preoperative (left) and postoperative (right). ( **A** ) Front views. ( **B** ) Oblique views. ( **C** ) Side views.

The 4Dx technique facilitates preoperative communication between the doctor and the patient. It objectively informs the patient all the areas that will be suctioned and the plan of surgery ( Figs. 6 7 8 ). This further helps set correct expectations on the outcomes of surgery. It is not only the breast region but the entire chest that is contoured, giving a more natural look to the patient. It also simplifies learning the procedure for beginners. It offers a comprehensive contemporary management of gynecomastia. However, there are a few pitfalls. It does not apply to the smallest grades of gynecomastia (grade 1) or even larger grades with predominantly gland component and very little fat. A study evaluating patient satisfaction scores is warranted in the future.

**Fig. 7:**
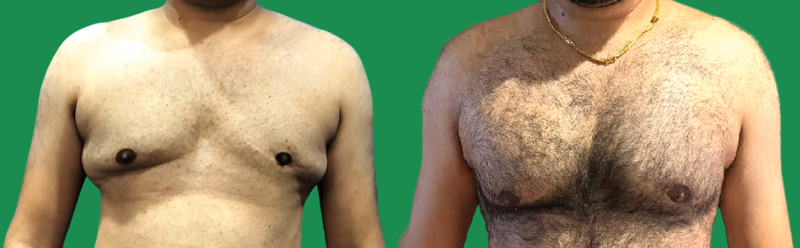
Another case of gynecomastia operated elsewhere, where only the gland removal and liposuction of surrounding areas was done. On photographic evaluation, it seemed like the patient has a lot of excess skin (left). However, on clinical examination, it was noted that the folds of the skin, especially on the sides had a lot of fat. The patient was treated as a grade 3a gynecomastia. With only the 4Dx technique of liposuction of the areas of concern, a well-balanced look of the chest was achieved at 3 months (right).

**Fig. 8:**
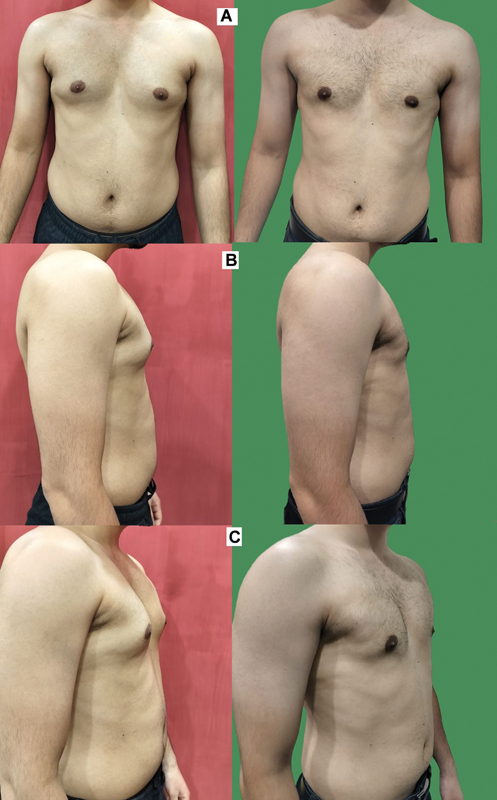
Preoperative (left) and postoperative (right) photographs of a patient with grade 2a gynecomastia operated with the 4Dx technique of liposuction. ( **A** ) Front views. ( **B** ) Side views. ( **C** ) Oblique views.

Thus, the 4Dx technique for liposuction in gynecomastia achieves liposuction in two perpendicular directions through two minimally scarring access points and possible individualization.
